# Symptom profiles of community cases infected by influenza, RSV, rhinovirus, seasonal coronavirus, and SARS-CoV-2 variants of concern

**DOI:** 10.1038/s41598-023-38869-1

**Published:** 2023-08-02

**Authors:** Cyril Geismar, Vincent Nguyen, Ellen Fragaszy, Madhumita Shrotri, Annalan M. D. Navaratnam, Sarah Beale, Thomas E. Byrne, Wing Lam Erica Fong, Alexei Yavlinsky, Jana Kovar, Susan Hoskins, Isobel Braithwaite, Robert W. Aldridge, Andrew C. Hayward

**Affiliations:** 1 MRC Centre for Global Infectious Disease Analysis and NIHR Health Protection Research Unit in Modelling and Health Economics, Department of Infectious Disease Epidemiology, School of Public Health, Imperial College London, London, UK; 2 Centre for Public Health Data Science, Institute of Health Informatics, University College London, London, UK; 3 Institute of Epidemiology and Health Care, University College London, London, UK; 4 Department of Infectious Disease Epidemiology, London School of Hygiene and Tropical Medicine, London, UK

**Keywords:** Signs and symptoms, Fatigue, Fever, Respiratory signs and symptoms, Respiratory tract diseases, Diseases, Infectious diseases, Viral infection

## Abstract

Respiratory viruses that were suppressed through previous lockdowns during the COVID-19 pandemic have recently started to co-circulate with SARS-CoV-2. Understanding the clinical characteristics and symptomatology of different respiratory viral infections can help address the challenges related to the identification of cases and the understanding of SARS-CoV-2 variants' evolutionary patterns. Flu Watch (2006–2011) and Virus Watch (2020–2022) are household community cohort studies monitoring the epidemiology of influenza, respiratory syncytial virus, rhinovirus, seasonal coronavirus, and SARS-CoV-2, in England and Wales. This study describes and compares the proportion of symptoms reported during illnesses infected by common respiratory viruses. The SARS-CoV-2 symptom profile increasingly resembles that of other respiratory viruses as new strains emerge. Increased cough, sore throat, runny nose, and sneezing are associated with the emergence of the Omicron strains. As SARS-CoV-2 becomes endemic, monitoring the evolution of its symptomatology associated with new variants will be critical for clinical surveillance.

## Introduction

In February 2022, the UK government announced its new strategy for “living with COVID-19”^1^. Most countries took a similar stance soon after. With the ending of all mandatory non-pharmaceutical interventions (NPIs) and subsequent increases in mixing patterns, respiratory viruses that were suppressed during previous lockdowns of the COVID-19 pandemic have recently started to co-circulate with SARS-CoV-2^2,3^. The US Centre for Disease Control and prevention (CDC) estimated that, as of 9 December 2022, flu related hospitalisations and deaths already surpassed those of the 2021–2022 flu-season^4,5^. Additionally, the recent wave of respiratory syncytial virus (RSV) cases raised concerns across Europe amid increased hospitalisations and deaths amongst children^6^. This, combined with decreased availability of free testing, will pose diagnostic challenges, which is important when considering syndromic surveillance and pharmaceutical management of SARS-CoV-2 and other respiratory viruses. Yet, there is a lack of community-based studies that have compared the frequency of symptoms between SARS-CoV-2 and other common respiratory viruses to identify potential differences in their symptomatology. Additionally, the emergence of multiple variants of concern (VOC) during the SARS-CoV-2 pandemic has led to uncertainties about potential changes in the symptomatology of COVID-19 illnesses. Several community-based studies have compared the symptoms of SARS-CoV-2 variants during the 1st wave of the pandemic^7–9^ and during the first 2 Omicron waves (Omicron BA1 and BA2)^10^. We provide more recent estimates by including Omicron BA5 illnesses up to September 2022.

This paper describes the symptoms of a range of respiratory infections experienced by confirmed cases from the Flu Watch Community Cohort (2006–2011) and the Virus Watch Cohort (2020–2022). We assessed how SARS-CoV-2 symptoms have changed with waves of different variants. We also assessed the applicability of World Health Organization (WHO) definitions of influenza-like illness (ILI) and acute respiratory infection (ARI) for the identification of different respiratory viruses amongst people with symptomatic disease.

## Methods

Flu Watch (2006–2011) and Virus Watch (2020–2022) are household community cohort studies investigating the epidemiology, clinical features, risk factors for transmission and immunity to influenza and SARS-CoV-2 respectively. Both cohorts recruited all members of participating households and followed them up with weekly surveys commencing on symptom occurrence.

### Flu Watch

Flu Watch was a household community cohort study following up entire households across England during six influenza seasons including three periods of seasonal influenza (winters 2006–2007, 2007–2008 and 2008–2009) and the first three waves of the 2009 influenza pandemic (summer 2009, autumn‐winter 2009/2010 and winter 2010/2011). 5 484 participants were followed up for 118 158 person‐weeks^11^. Participants were asked to submit nasal swabs on day two of any illness that was a “cough, cold, sore throat, or flu-like illness”. These were examined using polymerase chain reaction (PCR). All samples were analysed for influenza infection. At different times in the study, rhinovirus, RSV and seasonal coronavirus were also tested for using PCR. Only respiratory and constitutional symptoms were considered for this analysis (i.e. sore throat, cough, runny nose, sneezing, fever, headache, fatigue). Full details of the Flu Watch methodology have been previously published^12^.

### Virus Watch

Virus Watch is a household community cohort study following up entire households in England and Wales since mid-June 2020. By February 2022, 58 566 individuals in 28 495 households had registered to take part in the study. Participants submitted weekly online surveys reporting the date of any respiratory, constitutional, gastrointestinal, ocular, or skin symptoms experienced and the dates and results of any testing for SARS-CoV-2 by lateral flow test (LFT) or PCR. Symptoms included for the analysis were: cough, dry cough, sore throat, blocked nose, runny nose, sneezing, sinus pain, fever, fatigue, headache, muscle ache, loss of smell and loss of taste. The Virus Watch study protocol is available online^13^.

### Data processing

For both studies, symptom data were extracted and grouped into illness episodes. Tests with no symptoms reported were excluded in both studies. The start date of an illness episode was defined as the first day any symptoms were reported, and the end date was the final day of reported symptoms. A seven-day washout period in which no symptoms were reported was used to identify separate illness episodes.

In Virus Watch, swab results were matched to illnesses that were within seven days of the illness start date. In addition to self-reported SARS-CoV-2 test results, test results from the UK Second-Generation Surveillance System (SGSS) dataset were linked to the Virus Watch dataset. Participant data were linked over time and between databases using the unique personal identifier recorded at all interactions with the English National Health Service (NHS), full name, date of birth and home address. SGSS contains SARS-CoV-2 test results during Pillar 1 (testing patients in secondary care) and Pillar 2 (community testing).

### SARS-CoV-2 variant designation

Testing (by LFT or PCR) detects the presence of SARS-CoV-2 but, unlike RNA sequencing, does not identify the variant. Therefore, using national surveillance data^14^, we designated a variant to a case if that variant was making up at least 75% of all regional sequenced genomes at the time of the case’s symptom onset. Illnesses in regions and weeks that did not have a dominant variant reaching at least 75% of all sequenced genomes were excluded. Variants are defined as per the UK Health Secretary Agency (UKHSA) definition and wild-type refers to all SARS-CoV-2 variants circulating before the Alpha variant^14^.

### Analysis

We present symptom profiles of individuals with confirmed SARS-CoV-2 (n = 10 986), seasonal coronavirus (HCoV-NL63, HCoV-OC43, and HCoV-229E) (n = 191), influenza (n = 222), RSV (n = 84) and rhinovirus (n = 283) and compare the proportion of symptoms experienced during illnesses.

We also compare the compatibility of the WHO case definition for ARI (sudden onset of symptoms with cough and/or sore throat and/or runny nose) and WHO case definition for ILI (fever >  = 38 °C and cough).

As a sensitivity analysis to account for the fact that Flu Watch samples were submitted from anyone with cough, sore throat, cold or flu-like illness, whereas the national COVID-19 testing program encouraged submission of samples from all those with persistent cough, fever or loss of sense of smell or taste, we restricted SARS-CoV-2 cases to those submitted from participants who met the definition for ARI (Appendix 1).

We ran multiple multivariate logistic regressions to describe the odds of experiencing different respiratory and constitutional symptoms by SARS-CoV-2 VOC. Our primary exposure was SARS-CoV-2 variant strain (wild-type, Alpha, Delta, Omicron BA1, Omicron BA2) with Omicron BA5 as variant of reference. We controlled for known confounders reported in the literature including sex, age, clinal vulnerability and natural or vaccine induced immunity^15–17^. Clinical vulnerability status was derived from self-reported data on immunosuppressive therapy, cancer diagnoses, and chronic disease status. Participants were considered to have past exposure (and therefore some degree of immunity) to SARS-CoV-2 if they reported a SARS-CoV-2 positive illness or vaccination between 90- and 14- days prior symptom onset. This time window was based on the UK Health Security Agency (UKHSA) and the European Centre for Disease Control (ECDC) reports on SARS-CoV-2 immune response and immunity^15,17^.

### Ethics

The Virus Watch study was approved by the Hampstead NHS Health Research Authority Ethics Committee. Ethics approval number—20/HRA/2320. All members of participating households provided informed consent for themselves and, where relevant, for children that they were responsible for. This was electronically collected during registration. All necessary patient/participant consent has been obtained and the appropriate institutional forms have been archived. All methods were performed in accordance with the relevant guidelines and regulations.

## Results

### Flu Watch vs. Virus Watch

**Figure 1:**
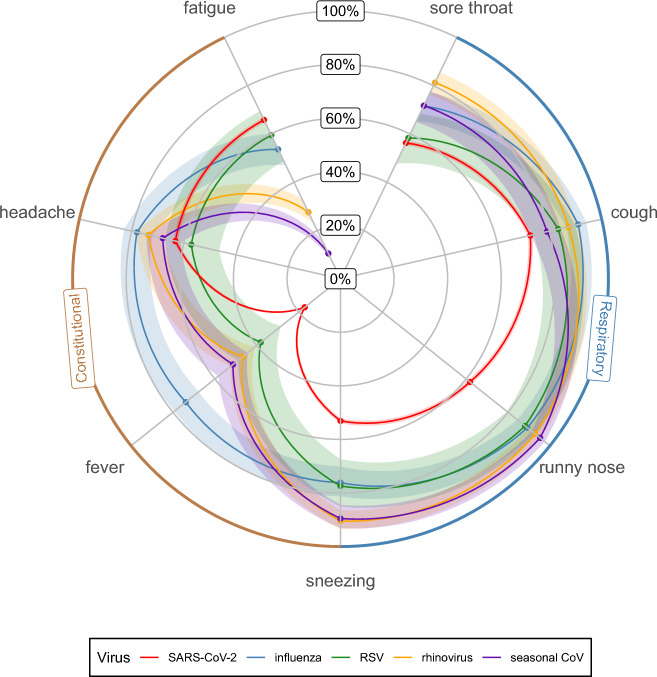
Symptom profile of common respiratory viruses: frequency of symptoms reported during illness by virus type (points represent the mean estimates, shaded areas represent the 95% confidence intervals (CI)). SARS-CoV-2 includes the wild-type, Alpha, Delta, Omicron BA1, Omicron BA2 and Omicron BA5 variants.

The frequency of symptoms of SARS-CoV-2 and for other respiratory viruses are shown in Table 1 (for every SARS-CoV-2 VOC) and illustrated in the radar plot in Fig. 1 (aggregated across all SARS-CoV-2 VOC).

**Table 1:** Frequency of symptoms reported during illness by virus type [95% confidence interval].

Characteristic	Overall, N = 11,766^1^	Wild-type, N = 262 [95% CI]^1,2^	Alpha, N = 445 [95% CI]^1,2^	Delta, N = 1,640 [95% CI]^1,2^	Omicron BA1, N = 2,282 [95% CI]^1,2^	Omicron BA2, N = 4,012 [95% CI]^1,2^	Omicron BA5, N = 2,345 [95% CI]^1,2^	Influenza, N = 222 [95% CI]^1,2^	Rhinovirus, N = 283 [95% CI]^1,2^	RSV, N = 84 [95% CI]^1,2^	Seasonal CoV, N = 191 [95% CI]^1,2^
Sore throat	57%	40% [34, 46]	39% [34, 43]	43% [40, 45]	53% [51, 55]	62% [61, 64]	65% [63, 66]	72% [65, 77]	81% [76, 86]	58% [47, 69]	72% [65, 78]
Cough	74%	65% [58, 70]	60% [55, 65]	65% [63, 67]	61% [59, 63]	79% [78, 81]	81% [79, 82]	91% [86, 94]	87% [83, 91]	83% [73, 90]	79% [72, 84]
Runny nose	64%	35% [30, 42]	36% [31, 40]	57% [54, 59]	57% [55, 59]	71% [69, 72]	63% [61, 65]	89% [84, 93]	93% [89, 96]	88% [79, 94]	95% [91, 98]
Sneezing	55%	36% [30, 42]	37% [32, 42]	47% [44, 49]	49% [47, 51]	60% [59, 62]	55% [53, 57]	76% [70, 81]	90% [86, 94]	77% [67, 86]	90% [84, 93]
Fever	20%	23% [18, 28]	24% [20, 29]	23% [21, 25]	14% [12, 15]	14% [13, 15]	19% [18, 21]	74% [67, 79]	47% [41, 53]	38% [28, 49]	51% [44, 59]
Headache	64%	67% [61, 73]	65% [60, 69]	65% [63, 67]	61% [59, 63]	62% [60, 63]	66% [64, 68]	78% [72, 83]	73% [68, 78]	57% [46, 68]	68% [61, 75]
Fatigue	64%	71% [65, 77]	62% [57, 66]	64% [62, 67]	57% [55, 59]	68% [66, 69]	72% [70, 74]	54% [47, 60]	28% [23, 33]	60% [48, 70]	10% [6.7, 16]
ARI^3^	87%	78% [73, 83]	76% [71, 80]	81% [79, 83]	80% [79, 82]	91% [90, 91]	90% [89, 91]	99% [96, 100]	99% [97, 100]	100% [95, 100]	99% [97, 100]
ILI^4^	14%	17% [13, 22]	17% [13, 20]	17% [15, 19]	8.7% [7.6, 9.9]	12% [11, 13]	16% [15, 18]	49% [42, 55]	12% [8.3, 16]	20% [13, 31]	12% [7.5, 17]
Age											
0–15	11%	8.4% [5.5, 13]	9% [6.6, 12]	23% [21, 25]	15% [14, 17]	4.5% [3.9, 5.2]	3.2% [2.5, 4.0]	43% [36, 50]	19% [15, 25]	36% [26, 47]	20% [15, 26]
16–44	19%	35% [29, 41]	37% [33, 42]	27% [25, 29]	23% [22, 25]	14% [13, 16]	9.6% [8.4, 11]	23% [17, 29]	29% [24, 34]	16% [9.2, 26]	29% [23, 36]
45–64	39%	35% [30, 42]	38% [33, 43]	35% [33, 37]	35% [33, 37]	41% [40, 43]	43% [41, 45]	29% [23, 36]	36% [30, 42]	30% [20, 41]	38% [31, 45]
65 +	32%	21% [16, 27]	16% [13, 20]	15% [14, 17]	26% [24, 28]	40% [38, 41]	45% [42, 47]	5.5% [3.0, 9.6]	16% [12, 21]	19% [11, 29]	14% [9.2, 19]
Missing	5	0	0	0	0	0	0	2	0	3	0
Proportion	100%	2.2%	3.8%	14%	19%	34%	20%	1.9%	2.4%	0.7%	1.6%

^1^%.

^2^*CI *confidence interval.

^3^*ARI* acute respiratory infection (experiencing at least one of the following: cough, fever or runny nose).

^4^*ILI *influenza like illness (experiencing fever and cough).

Cough was most common amongst influenza, rhinovirus, RSV and SARS-CoV-2 Omicron BA5 illnesses with 91, 87, 83, 81 percent of illnesses reporting cough respectively. Cough was significantly less common in Alpha, Omicron BA1 and wild-type SARS-CoV-2 variants with respective frequencies of 60, 61 and 65% (Table 1).

Fever was significantly more common amongst influenza illnesses (74%, 95% CI:  68–79) compared to other respiratory viruses. On average, only 17% (16–18) of COVID-19 illnesses reported fever (Fig. 1). However, this proportion varied across SARS-CoV-2 variants, with the Omicron BA1 (14%, 12–15) and BA2 (14%, 13–15) strains having significantly lower proportions compared to other variants.

**Figure 2:**
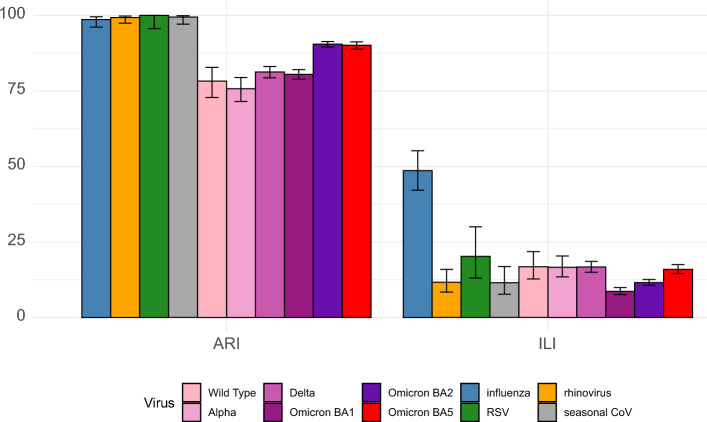
Proportions of illnesses meeting the WHO case definitions by virus type. Error bars represent 95% confidence intervals.

The frequency of illnesses meeting the ARI and ILI case definitions by viruses are displayed in Fig. 2. Nearly all influenza, RSV, rhinovirus and seasonal coronavirus illnesses met the ARI definition. Fewer SARS-CoV-2 illnesses met the ARI definition, with proportions ranging from 75.7% (71.5–79.5) for Alpha and 90.1% (88.8–91.3) for Omicron BA5. However, we observed a significant increase (+ 10 percentage points) in the proportion of Omicron BA2 and BA5 illnesses meeting the ARI case definition with respect to the previously circulating strains. The performance of ILI case definition was low with only 48.6% (42.2–55.2) of influenza illnesses meeting the case definition. Only 14.4% (11.5–18.2) of illnesses contracted from other respiratory viruses met the ILI case definition.

### Virus Watch

**Figure 3:**
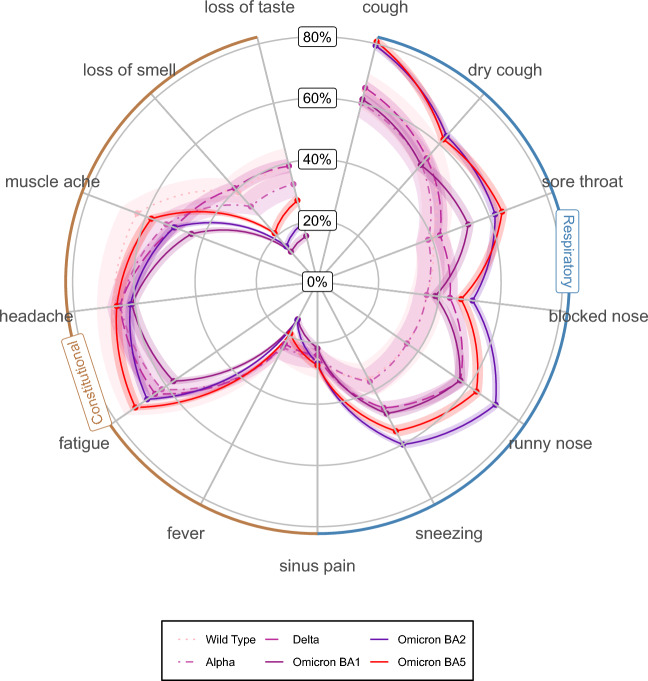
Symptom profile by SARS-CoV-2 VOC: frequency of symptoms reported during illness by VOC (points represent the mean estimates, shaded areas represent the 95% confidence intervals).

Figure 3 illustrates the frequency of symptoms during illnesses infected with all major SARS-CoV-2 VOC.

Over the course of the SARS-CoV-2 pandemic and with the emergence of new variants, there has been a gradual increase in the proportion of SARS-CoV-2 infections with upper respiratory symptoms (Table 1, Fig. 2). Since the Alpha variant became dominant in December 2020, COVID-19 illnesses have on average reported an increase in runny nose, sneezing and sore throat of six percentage points compared to previous strains. The increase in reported upper respiratory symptoms was particularly high when transitioning to Delta and Omicron BA2. The rise of the Delta variant, dominant in the UK from June 2021, was associated with a 11.7 percentage points increase in reported upper respiratory tract symptoms compared to Alpha illnesses. Finally, Omicron BA2 was associated with an average increase of 11.3 percentage points compared to illnesses infected with the BA1 strain. Overall, the percentage of illnesses reporting runny nose, sneezing and sore throat went from 35, 36, 40% respectively when wild-type was dominant to 71, 60, 62% under Omicron BA2 dominance. 65% of Omicron BA5 illnesses reported sore throat, 63% experienced runny nose and 55% experienced sneezing. Since the emergence of the Omicron strain, there has been a significant decrease in the proportion of SARS-CoV-2 cases reporting symptoms of loss of sense of smell or taste (40% for wild-type to 20% for Omicron BA5) (Fig. 3).

**Figure 4:**
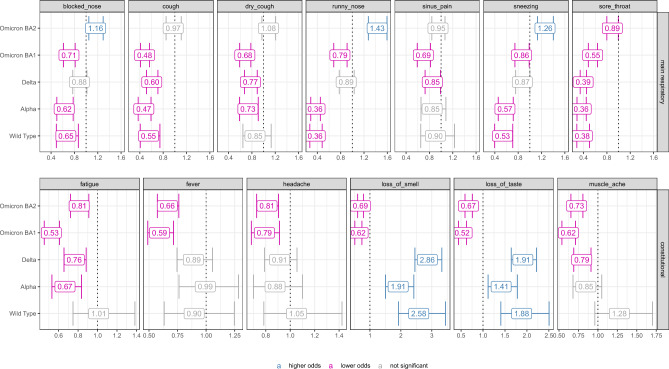
Odds ratios from multivariate logistic regressions with SARS-CoV-2 variant as primary exposure and Omicron BA5 as the variant of reference. Confounders including sex, age, clinal vulnerability and natural or vaccine induced immunity were controlled for.

Multivariate logistic regressions were used to describe the odds of experiencing respiratory and constitutional symptoms by SARS-CoV-2 VOC with Omicron BA5 as the reference variant, controlling for known confounders (Fig. 4). Compared to Omicron BA5, all previously circulating strains were generally associated with decreased odds of cough and sore throat, controlling for all other variables in the model. Infections with any pre-Omicron strain (Delta, Alpha, wild-type) decreased the odds of experiencing sore throat by over 60% (Odds Ratios (OR) 0.38, 95%CI: 0.30–0.46). Infections with Omicron BA 1 and Omicron BA 2 decreased the odds of experiencing sore throat by 45 and 11 percent respectively (0.55, 0.48–0.64; 0.89, 0.80–0.99). Omicron BA2 was associated with increased odds of sneezing (1.26, 1.13–1.40). Pre-Omicron strains were all associated with increased odds of loss of smell and loss of taste, with Delta showing the largest effect with odds ratios of 2.86 (2.45–3.33) and 1.91 (1.64–2.21) respectively. Compared to Omicron BA 5, all previously circulating strains at the exception of wild-type were associated with lower odds of experiencing fatigue with Omicron BA 1 showing the lowest odds ratios of 0.53 (0.45–0.60).

## Discussion

We describe the symptom profile for multiple respiratory viruses that are now co-circulating with SARS-CoV-2. Over time, the symptom profile of SARS-CoV-2 illnesses gradually became more similar to other respiratory viruses through increased frequency of cough, sneezing, runny nose and sore throat. Furthermore, the frequency of SARS-CoV-2 illnesses meeting the WHO ARI case definition has significantly increased with the Omicron BA2 and BA5 variants. Fever remains more common in influenza than other respiratory viruses studied. The growing similarity between symptoms of SARS-CoV-2 and other respiratory illnesses will make syndromic surveillance less effective, emphasising the importance of multi-pathogen virological surveillance. Continued use of diagnostic tests to distinguish between influenza and SARS-CoV-2 will be important for high-risk patients in whom antiviral medication is being considered.

Several factors could explain the observed changes in the SARS-CoV-2 symptomatology. The increase in cough and sneezing associated with the Omicron strains could contribute to their increased transmissibility compared to previous variants^18^, as these two symptoms are important pathways for expelling viral particles. This is in line with studies comparing the association of SARS-CoV-2 symptom presence, variants, and viral load^10,19,20^. Changing levels of immunity in the population during the pandemic through natural infections, vaccination and natural/vaccine induced immunity waning may also affect the symptomatology. Despite widespread vaccination and natural infections in our cohort (Appendices 2 and 3), the presence of respiratory symptoms has significantly increased at the same time as the Omicron strains spread. However, it is not clear whether observed change in symptomatology is due to the evolving SARS-CoV-2 variants or due to changing levels in immunity across the population.

Several community-based studies have compared the symptoms of SARS-CoV-2 variants during the 1^st^ wave of the pandemic^7–9^ and the first two Omicron waves^10^. Our results are in line with previous findings but provide additional information about Omicron BA5. A few studies^21–23^ published early in the pandemic qualitatively compared the symptoms of SARS-CoV-2 with other respiratory viruses but provided no quantitative insight about the symptom profiles of infected individuals. To our knowledge, no other study compared the frequency of symptoms experienced during influenza, RSV, rhinovirus, seasonal coronavirus, and SARS-CoV-2 VOC infections. Strengths of the study include the large number of participants in both Flu Watch and Virus Watch, the weekly reporting of symptoms and swab test results over multiple years, allowing to compare seasonal and endemic viral illnesses. Limitations of our analysis include the reliance on self-taken samples for Flu Watch and self-reported results for Virus Watch meaning that we would miss illnesses that did not test. However, most reported illness episodes in Virus Watch (56.3%) were tested for SARS-CoV-2 across the study period, with weekly proportions ranging from 4 to 91% (Appendix 4). Given that variants emerged at different times, time confounding variables such as “allergy seasons” could affect our results. However, our study spans over multiple years allowing us to compare variants that emerged during the same seasons (e.g. Alpha vs. Omicron BA1 and BA2, wild-type vs. Omicron BA5). We compared symptom frequencies of common respiratory viruses with SARS-CoV-2 based on two different time periods. It is important to note that the characteristics of respiratory infections for the same virus can vary across seasons and regions due to the prevalence of different types and subtypes^24^. We aggregated the FluWatch illnesses across multiple seasons in which different types and subtypes could have had different effects on symptomatology. The currently circulating strain of influenza (or RSV, rhinovirus, or seasonal coronavirus) in the UK might have different characteristics compared to the ones analysed from our 2006–2011 data. However, to our knowledge, there is no clear evidence of a significant change in influenza symptomatology in the year 2022 in the UK^24^. We do not assess the duration and severity of illness, which can substantially differ between the studied pathogens.

As SARS-CoV-2 VOC emerged, the SARS-CoV-2 symptomatology gradually resembled that of other respiratory symptoms. The more contagious Omicron strains were significantly associated with an increase in cough and sneezing. Untangling the potential relationships between symptomatology, immune mechanisms and viral genetic evolution could help us better understand the transmission advantages associated with the more recent SARS-CoV-2 variants.

## Supplementary Information

Supplementary Information.

## Data Availability

We aim to share aggregate data from this project on our website www.ucl-virus-watch.net and via a "Findings so far" section on our website. We are sharing individual record-level data on the Office of National Statistics (ONS) MDX Browser > Virus Watch—England and Wales @ 89201 (metadata.works). The data are available under restricted access as they contain sensitive health data. Access can be obtained by ONS Secure Research Service. The code for this analysis is available on GitHub https://github.com/CyGei/Symptom-profiles.

## References

[1] Gov.uk. COVID-19 Response: Living with COVID-19. In: Office C, editor. 2022.

[2] Kim J-H, Roh YH, Ahn JG, Kim MY, Huh K, Jung J (2021). Respiratory syncytial virus and influenza epidemics disappearance in Korea during the 2020–2021 season of COVID-19. Int. J. Infect. Dis..

[3] Hodjat P, Christensen PA, Subedi S, Bernard DW, Olsen RJ, Long SW (2021). The reemergence of seasonal respiratory viruses in Houston, Texas, after relaxing COVID-19 restrictions. Microbiol Spectr..

[4] CDC CfDCaP. Weekly U.S. Influenza Surveillance Report. 2022.

[5] CDC. Preliminary Estimated Influenza Illnesses, Medical visits, Hospitalizations, and Deaths in the United States – 2021–2022 influenza season. 2022 04 October 2022.

[6] Joint statement - Influenza season epidemic kicks off early in Europe as concerns over RSV rise and COVID-19 is still a threat [press release]. 01–12–2022 2022.

[7] Grant MC, Geoghegan L, Arbyn M, Mohammed Z, McGuinness L, Clarke EL (2020). The prevalence of symptoms in 24,410 adults infected by the novel coronavirus (SARS-CoV-2; COVID-19): A systematic review and meta-analysis of 148 studies from 9 countries. PLoS ONE.

[8] Menni C, Valdes AM, Polidori L, Antonelli M, Penamakuri S, Nogal A (2022). Symptom prevalence, duration, and risk of hospital admission in individuals infected with SARS-CoV-2 during periods of omicron and delta variant dominance: A prospective observational study from the ZOE COVID Study. Lancet.

[9] Mahase E (2021). Covid-19: Sore throat, fatigue, and myalgia are more common with new UK variant. BMJ.

[10] Whitaker M, Elliott J, Bodinier B, Barclay W, Ward H, Cooke G (2022). Variant-specific symptoms of COVID-19 in a study of 1,542,510 adults in England. Nat. Commun..

[11] Fragaszy EB, Warren-Gash C, White PJ, Zambon M, Edmunds WJ, Nguyen-Van-Tam JS (2018). Effects of seasonal and pandemic influenza on health-related quality of life, work and school absence in England: Results from the Flu Watch cohort study. Influenza Other Respir. Viruses.

[12] Fragaszy EB, Warren-Gash C, Wang L, Copas A, Dukes O, Edmunds WJ (2016). Cohort profile: The flu watch study. Int. J. Epidemiol..

[13] Hayward A, Fragaszy E, Kovar J, Nguyen V, Beale S, Byrne T (2021). Risk factors, symptom reporting, healthcare-seeking behaviour and adherence to public health guidance: Protocol for Virus Watch, a prospective community cohort study. BMJ Open.

[14] UKHSA. Investigation of SARS-CoV-2 variants: technical briefings. 2022.

[15] ECDC. Immune responses and immunity to SARS-CoV-2. European Center for Disewase Prevention and Control; 2021.

[16] Booth A, Reed AB, Ponzo S, Yassaee A, Aral M, Plans D (2021). Population risk factors for severe disease and mortality in COVID-19: A global systematic review and meta-analysis. PLoS ONE.

[17] UKHSA. UKHSA SARS-CoV-2 reinfection guideline: Investigation and management of suspected SARS-CoV-2 reinfections. 2021.

[18] Chen J, Wang R, Gilby NB, Wei G-W (2022). Omicron variant (B.1.1.529): Infectivity, vaccine breakthrough, and antibody resistance. J. Chem. Inform. Model..

[19] Stankiewicz Karita HC, Dong TQ, Johnston C, Neuzil KM, Paasche-Orlow MK, Kissinger PJ (2022). Trajectory of Viral RNA load among persons with incident SARS-CoV-2 G614 infection (Wuhan Strain) in association with COVID-19 symptom onset and severity. JAMA Netw. Open.

[20] Kidd M, Richter A, Best A, Cumley N, Mirza J, Percival B (2021). S-variant SARS-CoV-2 lineage B1.1.7 is associated with significantly higher viral load in samples tested by TaqPath polymerase chain reaction. J. Infect. Dis..

[21] Bai Y, Tao X (2021). Comparison of COVID-19 and influenza characteristics. J. Zhejiang Univ. Sci. B.

[22] Jiang C, Yao X, Zhao Y, Wu J, Huang P, Pan C (2020). Comparative review of respiratory diseases caused by coronaviruses and influenza A viruses during epidemic season. Microbes Infect..

[23] Pormohammad A, Ghorbani S, Khatami A, Razizadeh MH, Alborzi E, Zarei M (2021). Comparison of influenza type A and B with COVID-19: A global systematic review and meta-analysis on clinical, laboratory and radiographic findings. Rev. Med. Virol..

[24] Gov.uk. Official Statistics: Surveillance of influenza and other seasonal respiratory viruses in winter 2021 to 2022. 2022.

