# Supplementary material for: Symptom profiles of community cases infected by influenza, RSV, rhinovirus, seasonal coronavirus, and SARS-CoV-2 variants of concern
Source: Sci Rep. 2023 Aug 2;13:12511. doi: 10.1038/s41598-023-38869-1 (PMC10397315; doi:10.1038/s41598-023-38869-1)
Supplement: Supplementary file 1 — Supplementary Information. [file 41598_2023_38869_MOESM1_ESM.docx]

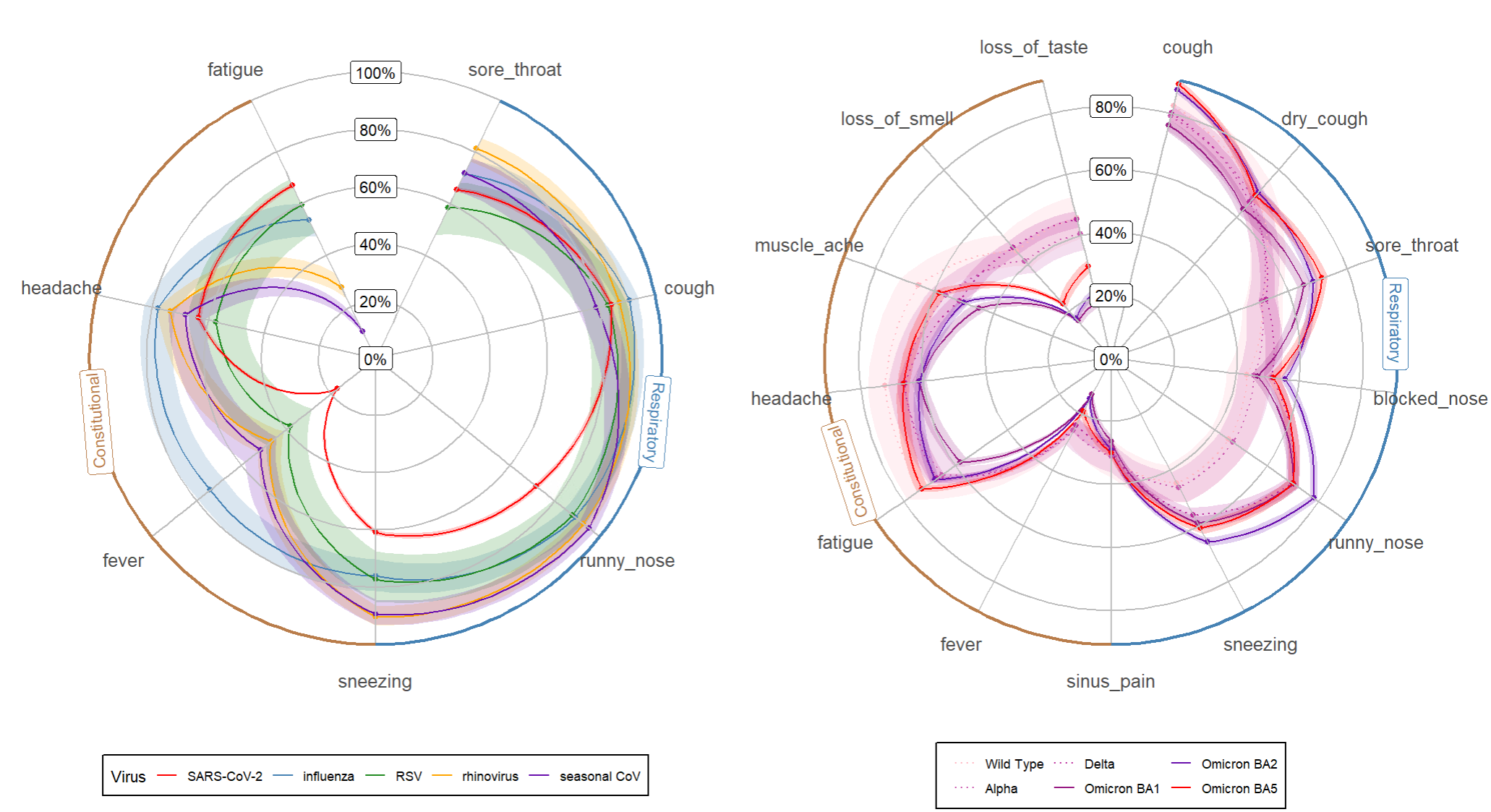


Appendix 1: Sensitivity analysis: Restricting SARS-CoV-2 illnesses meeting the ARI WHO case definition.


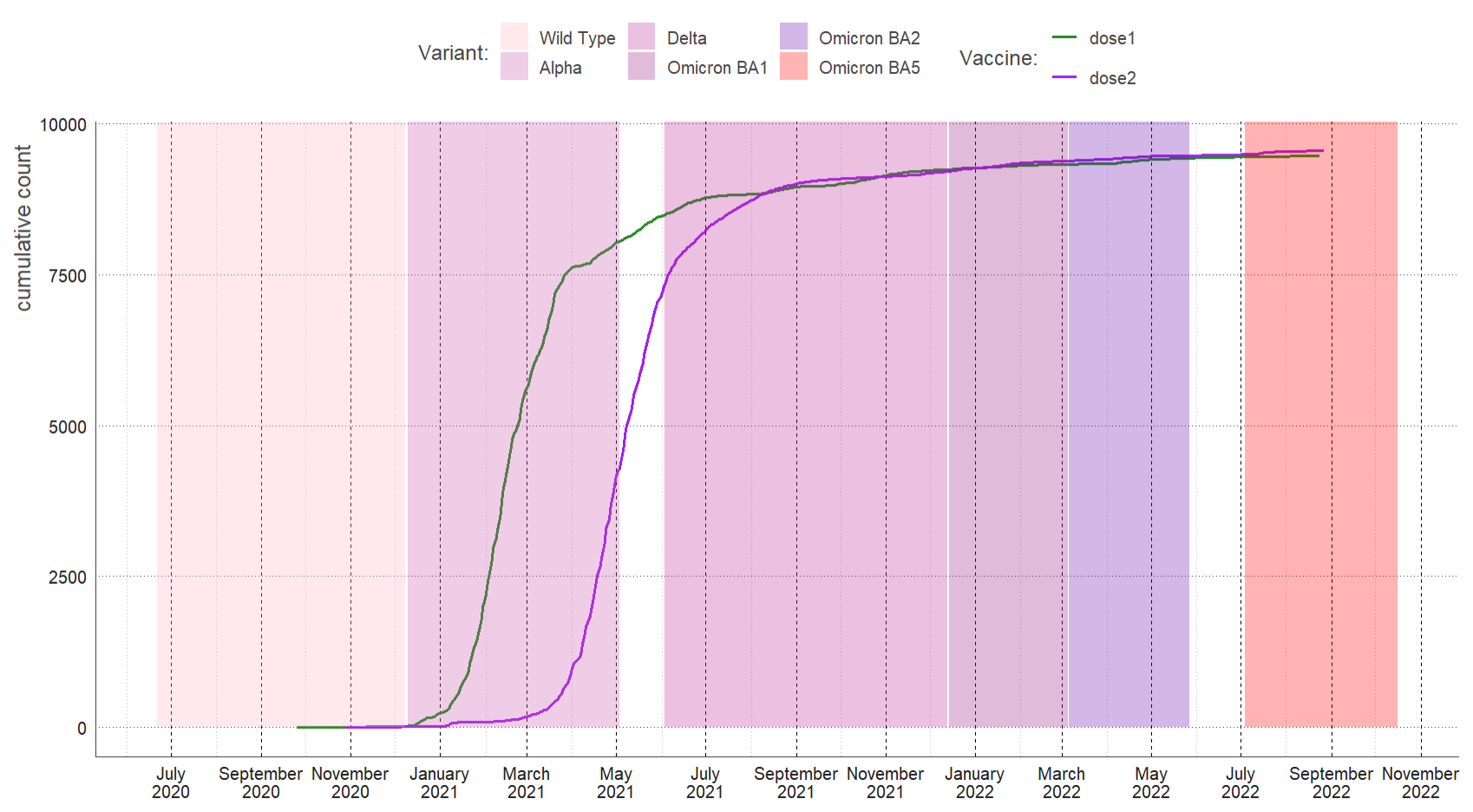


Appendix 2: Cumulative count of SARS-CoV-2 vaccination dose 1 and dose 2.


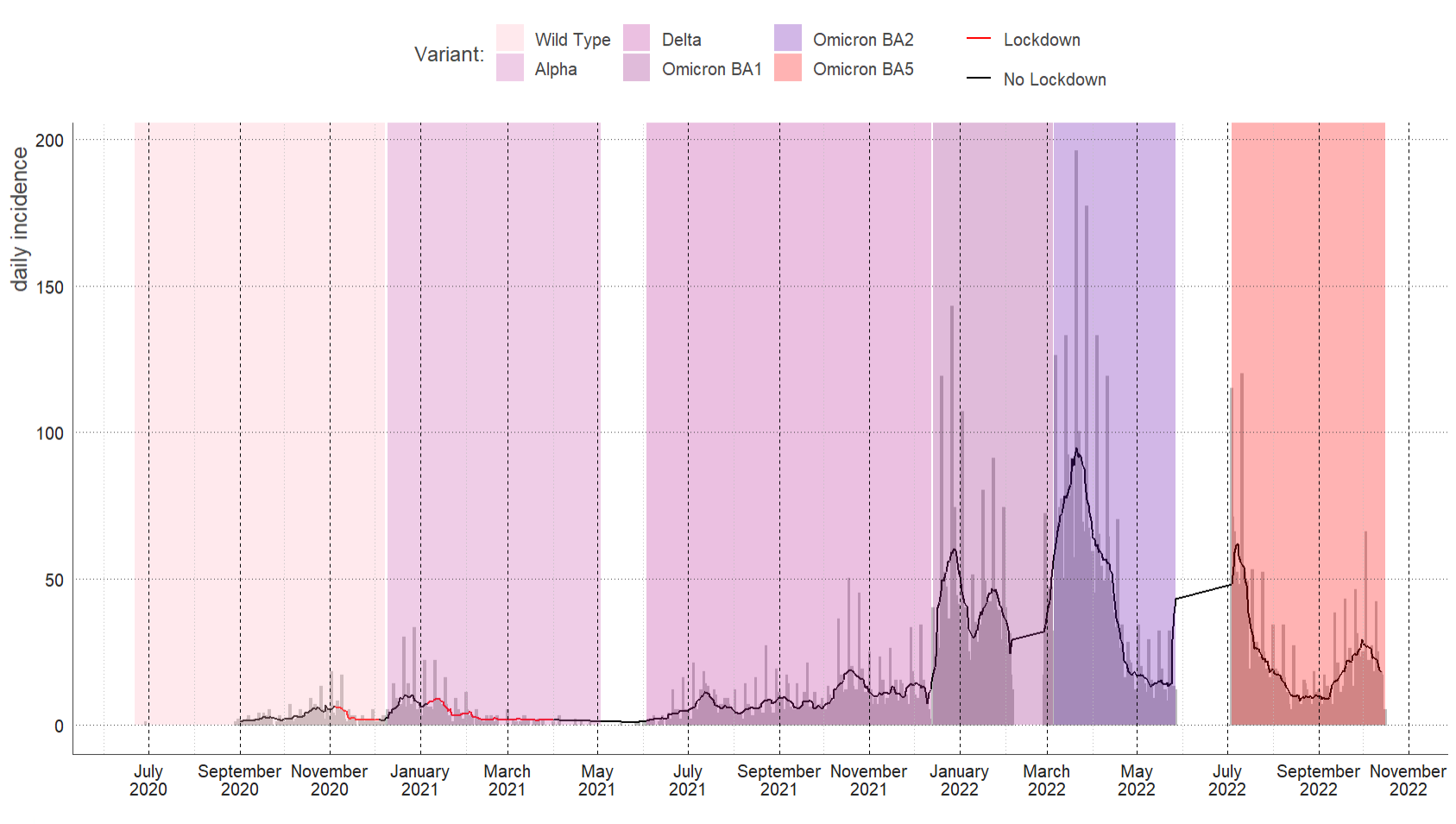


Appendix 3: proportion of SARS-CoV-2 positive illnesses (grey bars represent daily proportion, line represents 7 -day average).


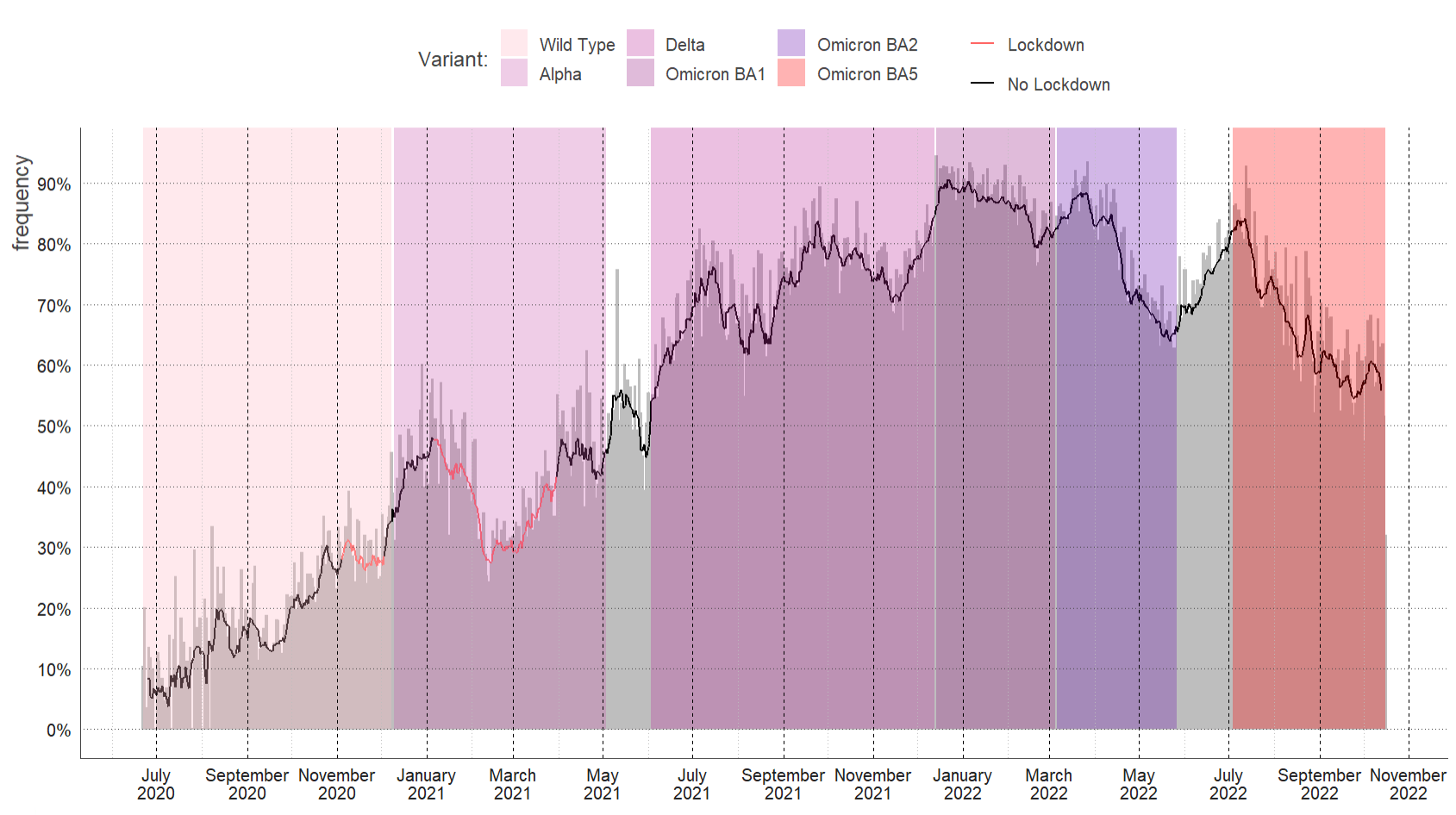


Appendix 4: proportion of illnesses tested for SARS-CoV-2 (grey bars represent daily proportion, line represents 7 -day average).
